# Supplementary material for: Colorectal cancer-associated SNP rs17042479 is involved in the regulation of NAF1 promoter activity
Source: PLoS One. 2022 Sep 6;17(9):e0274033. doi: 10.1371/journal.pone.0274033 (PMC9447907; doi:10.1371/journal.pone.0274033)
Supplement: Attachment — Submitted filename: Response to Reviewers.docx [file pone.0274033.s002.docx]

Dear reviewers.

Thank you very much for the comments. We have addressed the editorial comments 1-4. We have added our responses to the points and comments raised by the reviewers:

- Please ensure that your manuscript meets PLOS ONE's style requirements, including those for file naming. The PLOSONE style templates can be found at 
  <https://journals.plos.org/plosone/s/file?id=wjVg/PLOSOne_formatting_sample_main_body.pdf> and 
  [https://journals.plos.org/plosone/s/file?id=ba62/PLOSOne_formatting_sample_title_authors_affiliations.pdf](https://journals.plos.org/plosone/s/file?id=ba62/PLOSOne_formatting_sample_title_authors_affiliations.pdf)
  - We have gone through the style requirements and file naming.
- Please provide additional details regarding participant consent. In the ethics statement in the Methods and online submission information, please ensure that you have specified what type you obtained (for instance, written or verbal, and if verbal, how it was documented and witnessed). If your study included minors, state whether you obtained consent from parents or guardians. If the need for consent was waived by the ethics committee, please include this information.
  - We have added the information in the method section.
- We note that you have included the phrase “data not shown” in your manuscript. Unfortunately, this does not meet our data sharing requirements. PLOS does not permit references to inaccessible data. We require that authors provide all relevant data within the paper, Supporting Information files, or in an acceptable, public repository. Please add a citation to support this phrase or upload the data that corresponds with these findings to a stable repository (such as Figshare or Dryad) and provide and URLs, DOIs, or accession numbers that may be used to access these data. Or, if the data are not a core part of the research being presented in your study, we ask that you remove the phrase that refers to these data.
  - The phrase is removed from the manuscript.
- Please include your full ethics statement in the ‘Methods’ section of your manuscript file. In your statement, please include the full name of the IRB or ethics committee who approved or waived your study, as well as whether or not you obtained informed written or verbal consent. If consent was waived for your study, please include this information in your statement as well.
  - We have added the information in the method section.

Responses on points from reviewer 1:

- In abstract the conclusion lines should be clear and future driven. No such take home message is carried in the current abstract.
  - *We have added short conclusion in the abstract.*
- It would be better if the author can include the prediction analysis of the said new SIX SNPS as to how they will affect the functioning of the two genes Nuclear Assembly Factor 1 (NAF1) and Follistatin-Like 5 (FSTL5).
  - *We have analyzed for transcription factor binding site in a sequence around the SNP rs17042479 in the online tool Jaspar. We analyzed following sequence:* *AAACATGATTACACAGCCAT A/G GGGAACCTGGAGAAAAGGTT.*

*We scanned the sequences against all vertebrate matrix models in JASPAR. This resulted in a long list of possible transcription factor binding sites.* *However, we don’t believe it makes sense to present the list of potential transcription factors (of which many is not expressed the intestine) without a functional characterization.*

- Introduction section should add more information on the gene, their SNPs and the cross talk.
  - *We have added more information on the gene and the SNPs in the introduction, and added a figure with a zoom in of the SNPs in the risk locus in the result section.*
- Please provide either graphical representation of the SIX SNPS or tabulated form, showing the location of each SNP in the gene and what substitutions do occur in each. The current graphical representation (Figure 1a) is not good enough as well.
  - *We have extended figure 1a, and added a zoom in of the risk locus that presents a graphical representation of the six SNPs, furthermore the observed alleles are presented in the figure text.*
- The analysis of the cases based on grouping is also heterogeneous, which is important as it biases the results and interpretations implicating implicates that NAF1 as a tumor suppressor gene in CRC. Author need to make a clear explanation of this.
  - *We only identified one patient that was homozygous for the risk locus, so we have grouped this patient with heterozygote patients as we don’t believe it makes sense to present data for a single individual.*
- Author should exhaustively write the statistical analysis and test done. What were the descriptive and inferential statistics that were performed, need to be mention clearly?
  - *We have added more information about the statistical test used in the analysis in the method section.*
- It is not clear as to why Authors have kept four different values of P value as significant. The norm is always P<=0.05 is regarded as significant.
  - *It was not our intention to have four different values of P value as significant, just different symbols to show the P value. We have specified in the text in the method section, that we use P<=0.05 as a significant result.*
- Authors need to enlist all limitations of the study that might bias the interpretations at the end of the discussion.
  - *We have added a section with limitations in the end of the discussion.*

Responses on points from reviewer 2:

Major points

- A two-phase design would be more desirable and possibly, more robust results would have been drawn.
  - *Very good suggestion, but we are unfortunately not able to perform because the sample size is not big enough.*
- The authors should explain why B2M gene was chosen as reference gene. If they had used other housekeeping gene, maybe, presented results would have been more conclusive.
  - *Dydensborg et al.(PMID: 16399877), investigated potential reference genes for comparing human colon primary carcinomas with their corresponding normal mucosa and found that B2M was the best reference gene to compare diseased and healthy samples. We have added a sentence in the methods section about this.*
- An important issue is also the small size of the cohort which may limit study significance.
  - *We agree on this point, and we added a section about this problem in the discussion.*
- Based on the presented results, SNP rs17042479 (G) seems to have different impact in healthy compared to malignant tissues. This is a weird finding. How this observation could be explained?
  - *We have added a section discussing this observation in the discussion*
- Why the authors used B2M as a reference gene? Are there supportive data on CRC?
  - *We have explained this in question 2 regarding B2M.*
- Presented results regarding the associations of NAF1 expression with stage/differentiation seem to be arbitrary or borderline, especially if we take into consideration the number of patients included in stage IV and the extensive overlapping in boxplots (Fig 4).
  - *We agree on this point and decided to remove the results regarding the associations of NAF1 expression with stage/differentiation.*

Minor Points:

- Line 266-267: Please, this statement “Patients with the risk allele of SNP rs17042479 are heterozygous for the SNP, it is possible that the effect is more evident in patients that are homozygous for the risk allele of SNP rs17042479” should be clarified.
  - *We have rewritten the sentence to clarify.*
- Abbreviations should be expanded throughout the manuscript.
  - *We have checked the manuscript for abbreviations that is not expanded the first time it is mentioned.*
