# Supplementary material for: Colorectal cancer-associated SNP rs17042479 is involved in the regulation of NAF1 promoter activity
Source: PLoS One. 2022 Sep 6;17(9):e0274033. doi: 10.1371/journal.pone.0274033 (PMC9447907; doi:10.1371/journal.pone.0274033)
Supplement: Attachment — Submitted filename: Response to Reviewers.docx [file pone.0274033.s003.docx]

Dear editor and reviewer.

Thank you very much for the extra comments. We have checked the reference list to ensure that it is complete and correct.

Comments from reviewer 2:

The authors have adressed the majority of my comments with the exception of the comment regarding the different impact of SNP rs17042479 (G) in healthy compared to malignant tissues.
Although the authors have added a sentence in the discussion section, providing a possible explanation, however, they don't provide relevant refs which could support this explanation. This point needs to be treated further.

*We have elaborated more on this finding in the discussion and added references.*
